# Transcranial magnetic stimulation and amyloid markers in mild cognitive impairment: impact on diagnostic confidence and diagnostic accuracy

**DOI:** 10.1186/s13195-019-0555-3

**Published:** 2019-12-01

**Authors:** Alessandro Padovani, Alberto Benussi, Maria Sofia Cotelli, Clarissa Ferrari, Valentina Cantoni, Valentina Dell’Era, Rosanna Turrone, Barbara Paghera, Barbara Borroni

**Affiliations:** 1 Centre for Neurodegenerative Disorders, Neurology Unit, Department of Clinical and Experimental Sciences, Clinica Neurologica, University of Brescia, P.le Spedali Civili, 1, 25100 Brescia, Italy; 2 Neurology Unit, Ospedale Vallecamonica, Esine, Brescia, Italy; 3 Service of Statistics, IRCCS Centro San Giovanni di Dio Fatebenefratelli, Brescia, Italy; 4 Department of Neuroscience, Psychology, Drug Research and Child Health, University of Florence, Florence, Italy; 5 Nuclear Medicine Unit, Spedali Civili Brescia, Brescia, Italy

**Keywords:** Alzheimer disease, Frontotemporal lobar degeneration, Dementia with Lewy bodies, Mild cognitive impairment, Transcranial magnetic stimulation, Short-interval intracortical inhibition, Short-latency afferent inhibition, Diagnostic confidence, Biomarkers

## Abstract

**Background:**

The development of diagnostic tools capable of accurately identifying the pathophysiology of mild cognitive impairment (MCI) has become a crucial target considering the claim that disease-modifying treatments should be administered as early as possible in the disease course. Transcranial magnetic stimulation (TMS) protocols have demonstrated analytical validity in discriminating different forms of dementia; however, its value in daily clinical practice in MCI subjects is still unknown.

**Objective:**

To evaluate the clinical value of TMS compared to amyloid markers on diagnostic confidence and accuracy in MCI subjects, considering clinicians’ expertise.

**Methods:**

One hundred seven MCI subjects were included and classified as MCI-Alzheimer disease (MCI-AD), MCI-frontotemporal dementia (MCI-FTD), MCI-dementia with Lewy bodies (MCI-DLB), or MCI-other in a three-step process based on (i) demographic, clinical, and neuropsychological evaluation (clinical work-up); (ii) clinical work-up PLUS amyloidosis markers or clinical work-up PLUS TMS measures; and (iii) clinical work-up PLUS both markers. Two blinded neurologists with different clinical expertise were asked to express a diagnostic confidence for each MCI subgroup, and ROC curve analyses were performed at each step.

**Results:**

The addition of TMS markers to clinical work-up significantly increased the diagnostic confidence for MCI-AD (*p* = 0.003), MCI-FTD (*p* = 0.044), and MCI-DLB (*p* = 0.033) compared to clinical work-up alone, but not for MCI-other (*p* > 0.05). No significant differences between the add-on effect of TMS and the add-on effect of amyloid markers to clinical work-up were observed (*p* > 0.732), while the diagnostic confidence further increased when both markers were available. The greater the clinical expertise, the greater the flexibility in considering alternative diagnosis, and the greater the ability to modify diagnostic confidence with TMS and amyloid markers.

**Conclusions:**

TMS in addition to routine clinical assessment in MCI subjects has a significant effect on diagnostic accuracy and confidence, comparable to well-established biomarkers of amyloidosis.

## Background

Diagnosis of mild cognitive impairment (MCI) relies on extensive evaluation of cognitive and behavioral performances, and refers to subjects with objective cognitive impairment with only minimal impairment in instrumental activities of daily living, who do not meet the criteria for dementia [1]. In about 20–40% of the cases, MCI represents the prodromal phase of Alzheimer disease (MCI-AD) [2, 3]. However, classification of MCI is complicated by the fact that it may be due either to metabolic disorders or to other neurodegenerative disorders, such as preclinical frontotemporal dementia (MCI-FTD) or preclinical dementia with Lewy bodies (MCI-DLB), or causes not related to progressive neurodegenerative diseases [1].

Thus, diagnosing the underlying etiology is challenging in an individual patient, and there is a need for accurate diagnostic tests and evidence of amyloid- and tau-related biomarkers.

In fact, clinical criteria state that positivity of one or more biomarkers of brain amyloidosis is associated with a high likelihood of AD in MCI subjects [4]. Decreased levels of Aβ_1-42_ in the cerebrospinal fluid and/or increased binding of amyloid brain imaging ligands on positron emission tomography are the most established and validated amyloid markers [5–8], being helpful in increasing the diagnostic confidence in patients with AD among clinicians [9, 10].

Our group has recently developed an index using transcranial magnetic stimulation (TMS) intracortical connectivity measures [11] that stemmed from the evidence that neurodegenerative dementias are characterized by a dysfunction of specific neurotransmitter circuits [12]. An impairment in cholinergic function has been widely reported in patients with AD and in DLB patients [13], whereas it has been demonstrated that GABAergic and glutamatergic interneurons are impaired in FTD and DLB [12, 14].

We measured short-latency afferent inhibition (SAI), a TMS paired-pulse protocol which indirectly and partially estimates the function of cholinergic circuits, and short-interval intracortical inhibition (SICI) and intracortical facilitation (ICF), markers which partially reflect GABA_A_ergic and glutamatergic neurotransmission, respectively [15]. By using SAI and SICI-ICF, we reported high accuracy values in identifying AD patients, even in the MCI stage [11, 16], as well as patients with FTD [11, 17, 18] or DLB [19]. Furthermore, we showed that TMS measures, when used on clinical grounds, increase diagnostic confidence of AD, comparable to that reported with established amyloidosis biomarkers [20].

However, in comparison to amyloid markers, TMS has its advantages: it is much less expensive, easy to perform, non-invasive, time saving, and safe.

However, despite the proven usefulness of both amyloid and TMS markers, all published studies have generally included selected research populations not representative of daily clinical practice, thus hampering the use of these markers. Moreover, to our knowledge, none of the available studies has assessed the role of clinicians’ expertise in the use of diagnostic markers on clinical grounds and how this influences the diagnostic confidence when markers are available.

All the above observations defined the objective of this work, aimed at evaluating the clinical utility of TMS compared to amyloid markers on diagnostic accuracy and confidence in subjects with MCI, taking clinicians’ expertise into consideration. To this end, we assessed the change of diagnostic confidence when either TMS intracortical connectivity measures or amyloid markers were randomly added to the routine clinical work-up, and eventually evaluated the impact when both markers were disclosed.

## Methods

### Participants and study design

Patients with MCI [1] were consecutively recruited from the Centre for Neurodegenerative Disorders and the Centre for Alzheimer Disease, University of Brescia, Brescia, Italy. Demographic characteristics, family history, and clinical features were carefully recorded. All patients considered in the present study underwent a standardized neuropsychological evaluation; brain magnetic resonance imaging; at least one diagnostic marker of brain amyloidosis, i.e., cerebrospinal fluid Aβ_1-42_ dosage and/or amyloid positron emission tomography scan; and TMS intracortical connectivity measures, as described below.

Patients’ data were then anonymized, randomized, and presented to two neurologists, one with long-lasting experience in a tertiary dementia care center (AP, rater 1) and one with 5-year experience in a secondary referral center for the diagnosis and the cure of dementia (MSC, rater 2), in three consecutive steps. In 50% of the cases (arm 1), the two raters were made aware of the following: step 1*—*demographic characteristics, family history, clinical and neuropsychological assessment, and structural imaging data (henceforth defined as “clinical work-up”); step 2*—*amyloid marker data; and step 3—TMS intracortical connectivity measures.

**Fig. 1:**
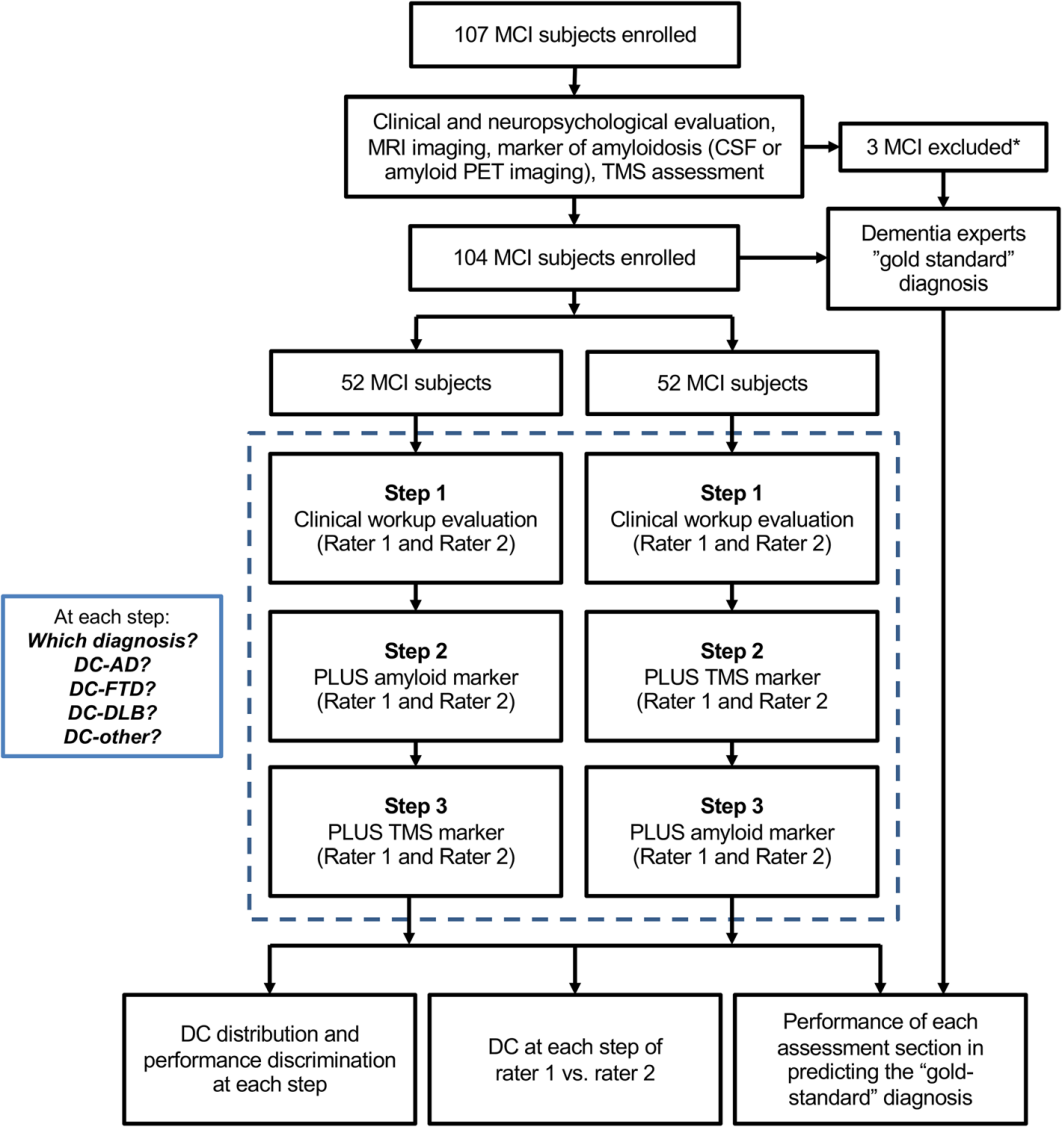
Study design. DC-AD, diagnostic confidence of mild cognitive impairment due to Alzheimer disease; DC-FTD, diagnostic confidence of mild cognitive impairment due to frontotemporal dementia; DC-DLB, diagnostic confidence of mild cognitive impairment due to dementia with Lewy bodies; DC-other, diagnostic confidence of mild cognitive impairment due to other conditions. *Excluded because carrying electronic implants (*n* = 2) or motor cortex excitability was unreliable (*n* = 1)

In the other 50% of the cases (arm 2), the two raters were made aware of the following: step 1—clinical work-up, step 2—TMS intracortical connectivity measures, and step 3—amyloid marker data (see Fig. 1, study design).

On the basis of the data obtained at each of the three steps, the two neurologists were asked to (a) formulate their etiological diagnosis (MCI-AD, MCI-FTD, MCI-DLB, or MCI-other), (b) to rate their diagnostic confidence (DC) that cognitive impairment was due to AD on a structured scale ranging from 0 to 100% (DC-AD, 0–100%), (c) to rate their confidence that cognitive impairment was due to FTD on a structured scale ranging from 0 to 100% (DC-FTD, 0–100%), (d) to rate their confidence that cognitive impairment was due to DLB on a structured scale ranging from 0 to 100% (DC-DLB, 0–100%),and (e) to rate their confidence that cognitive impairment was due to other neurodegenerative causes on a structured scale ranging from 0 to 100% (DC-other, 0–100%). Thus, the highest DC supported the formulated diagnosis. Any change in diagnosis or DC in the subsequent steps could only be attributed to knowing such results.

Moreover, a “gold standard” diagnosis (i.e., MCI-AD, MCI-FTD, MCI-DLB, or MCI-other) was provided by the dementia experts (AB, AA, and BB), who had the subjects in charge and who had complete access to all available information, such as the clinical work-up, amyloid markers, TMS intracortical connectivity measures, and follow-up evaluations.

#### Clinical work-up

The set of mandatory information for each recruited subject, which were presented to the two neurologists during the clinical work-up evaluation, included the demographic characteristics (age, sex, family history, past medical history, and comorbidities), the conventional structural brain imaging findings, and the results of the neuropsychological assessment, including global cognitive functions, long-term memory, executive functions, and language and visual spatial abilities, as previously reported [20]. Mini-Mental State Examination and Clinical Dementia Rating scales were considered to test global cognitive functions [21, 22]. The Basic and Instrumental Activities of Daily Living [23, 24], Neuropsychiatric Inventory [25], and Geriatric Depression Scale [26] were also considered.

All the above data were provided to the two raters in step 1.

#### Amyloid markers

We considered cerebrospinal Aβ_1-42_ analysis or amyloid positron emission tomography imaging as markers of amyloidosis. Lumbar puncture was carried out in the outpatient clinic according to standard procedures, and cerebrospinal fluid analysis was performed using an ELISA assay (INNOTEST, Innogenetics, Ghent, Belgium) [27]. According to our internal cutoff scores, a cerebrospinal fluid AD-like profile was defined as cerebrospinal fluid Aβ_1-42_ ≤ 650 pg/mL (along with cerebrospinal fluid total Tau ≥ 400 pg/mL).

Amyloid positron emission tomography imaging was acquired using 370 MBq (10 mCi) of ^18^F-florbetapir or ^18^F-flutemetamol, and visual readings were performed by a nuclear medicine physician who was blinded to the patients’ diagnosis, following the procedures provided by the ligand manufacturer, as previously reported [9].

Cerebrospinal fluid Aβ_1-42_ dosage (along with Tau) and/or amyloid positron emission tomography results (“positive” vs. “negative”) were provided to the two raters in either step 2 or step 3, according to randomization.

#### Transcranial magnetic stimulation intracortical connectivity measures

TMS protocols were carried out as previously published [11]. We considered SICI [28] and ICF [29], which predominantly reflect GABA_A_ergic and glutamatergic neurotransmission, respectively [15], and SAI [30], which primarily reflects cholinergic transmission [15].

Briefly, SICI, ICF, and SAI were studied using a paired-pulse technique, employing a conditioning-test design. For all paradigms, the test stimulus was adjusted to evoke a motor evoked potential (MEP) of approximately 1 mv amplitude in the right first dorsal interosseous muscle.

For SICI and ICF, the conditioning stimulus was adjusted at 70% of the resting motor threshold (RMT), employing multiple interstimulus intervals (ISIs), including 1, 2, 3, and 5 ms for SICI and 7, 10, and 15 ms for ICF [11, 28, 29]. SAI was evaluated employing a conditioning stimulus of single pulses (200 μs) of electrical stimulation delivered to right median nerve at the wrist, using a bipolar electrode with the cathode positioned proximally, at an intensity sufficient to evoke a visible twitch of the thenar muscles [11, 30]. Different ISIs were implemented (− 4, 0, + 4, + 8 ms), which were fixed relative to the N20 component latency of the somatosensory evoked potential of the median nerve.

For each ISI and for each protocol, 10 different paired conditioning-target stimuli and 14 control target stimuli were delivered in all participants in a pseudo-randomized sequence, with an intertrial interval of 5 s (± 10%). Stimulation protocols were conducted in a randomized order. All of the participants were capable of following instructions and reaching complete muscle relaxation; if, however, the data was corrupted by patient movement, the protocol was restarted and the initial recording was rejected.

The operators who performed TMS (VC and VD) were blinded to the subjects’ amyloid marker status and clinical or neuropsychological evaluation. Mean SICI-ICF (1, 2, 3 ms/7, 10, 15 ms) and mean SAI (0, ^+^4 ms), as well as SICI-ICF/SAI ratio, were calculated, as previously reported [11]. SICI-ICF/SAI ratio was provided to the two raters, and they considered the previous published cutoff value of 0.98 [11] in either step 2 or step 3, according to randomization.

### Statistical analysis

Sociodemographic characteristics of the patients as well as descriptive features of the DCs were provided through mean, standard deviation, 95% confidence intervals (95% CI), and median values.

Considering the experimental design (with repeated measures within arms, raters, and assessments and, thus, with variance structure dependence), and taking into consideration the diagnostic confidence distributions (skewed and with a positive mass at zero) of the four outcomes (DC-AD, DC-FTD, DC-DLB, DC-other), generalized estimating equation models with Tweedie distribution and log link-function were adopted to assess the association of the three factors: arms (arm 1 [clinical work-up➔amyloid markers➔TMS], arm2 [clinical work-up➔TMS➔amyloid markers]), raters (rater1, rater2), and single assessments (clinical work-up, TMS, amyloid markers) with DC. A first evaluation of the four DCs data with respect to arms, raters, and assessments was provided regardless of the diagnosis, by performing three generalized estimating equation models with DC as dependent variable and each of the three factors, and their triple interaction, as independent factors. Subsequently, a detailed evaluation of the additional contribution of the assessments (clinical work-up, clinical work-up PLUS either TMS or amyloid markers, and clinical work-up PLUS both markers) in explaining the DC variability was performed for each of the four diagnoses (MCI-AD, MCI-FTD, MCI-DLB, or MCI-other).

Finally, the association of DC of each of the five sections (independent variables) with the “gold-standard” diagnosis (i.e., MCI-AD, MCI-FTD, MCI-DLB, and MCI-other as, in turn, dependent variables) was evaluated through logistic regression models. Performance of each assessment section in predicting the “gold-standard” diagnosis was evaluated through receiver operating characteristic (ROC) curves, and the corresponding area under the curve (AUC) values, applied on predictive probability scores obtained by the logistic models. High values of AUC (greater than 0.8) indicate good performance of independent variables in predicting the diagnosis. Comparison of AUC was performed by the DeLong test.

Statistical significance was assumed at *p* < 0.05. Data analyses were carried out by “mclust” and “InformationValue” packages of R statistical software (URL http://www.R-project.org/) and IBM SPSS Statistics for Windows, version 21.0, Armonk, NY: IBM Corp.

## Results

### Participants

One hundred seven MCI subjects were consecutively enrolled in the present study. Three out of 107 were excluded (2.8%), because carrying electronic implants (*n* = 2) or motor cortex excitability was unreliable (*n* = 1).

Among 104 MCI subjects included in the present study, 52 (50%) were female, the mean age was 68.8 (standard deviation, 7.2), the mean age at onset was 65.4 (9.4), and the mean years of education was 10.3 (4.7). The mean Mini-Mental State Examination score was 26.5 (2.1), the mean Neuropsychiatry Inventory score was 8.8 (8.1), and the mean Geriatric Depression Scale score was 3.2 (3.1).

Forty-five MCI subjects (43.3%) performed positron emission tomography amyloid, 45 (43.3%) underwent lumbar puncture and cerebrospinal fluid analysis, and 14 (13.4%) performed both.

### Diagnostic confidence: description of the four DC outcomes and association with arm and raters’ clinical expertise

Descriptive statistics (mean and corresponding 95% CI, and median values) of the four outcomes are reported in Additional file 1: Figure S1. The DC distributions were extremely positively skewed (except for DC-AD in which the positive mass at zero was less marked). Overall, generalized estimating equation estimated mean for DC-AD was 45.3 (95% CI 40.0–51.1, median = 40), for DC-FTD was 28.9 (95% CI 24–34.8, median = 20), for DC-DLB was 9.8 (95% CI 6.8–14.3, median = 0), and for DC-other was 16.5 (95% CI 12.8–21.4, median = 0).

The evaluation of the DC in terms of different arms, raters, and assessments was provided regardless the diagnosis, by performing generalized estimating equation models with the four DCs, in turn, as dependent variable and each of the three factors as independent factors.

No evidence of statistically significant association between arm (arm 1: clinical work-up➔amyloid markers➔TMS vs. arm 2: clinical work-up➔TMS➔ amyloid markers) and the four DCs (*p* = 0.231, *p* = 0.184, *p* = 0.148, and *p* = 0.194 for DC-AD, DC-FTD, DC-DLB, and DC-other respectively) was found.

When the performance of rater 1 and rater 2 was considered, a significant difference for MCI-FTD, MCI-DLB, and MCI-other (*p* = 0.002, 0.003, 0.046, respectively), and a tendency toward significance (*p* = 0.095) for MCI-other, was found (see Additional file 2: Figure S2). The greater the clinical expertise, the greater the flexibility in considering alternative diagnosis other than MCI-AD after clinical work-up evaluation and the greater the ability to interpret TMS and amyloid markers by changing DC was documented. Thus, the rater with less experience (rater 2) showed more unwillingness to modify the first DC, based on clinical work-up, during the additional assessments, as compared to the rater with more experience.

### Diagnostic confidence of MCI-AD, MCI-FTD, MCI-DLB, and MCI-other of TMS and amyloid markers

A detailed evaluation of additional contribution at each step of the assessments (clinical work-up, clinical work-up PLUS either TMS or amyloid markers, clinical work-up PLUS both markers) in explaining the DC variability was performed within each diagnosis.

**Fig. 2:**
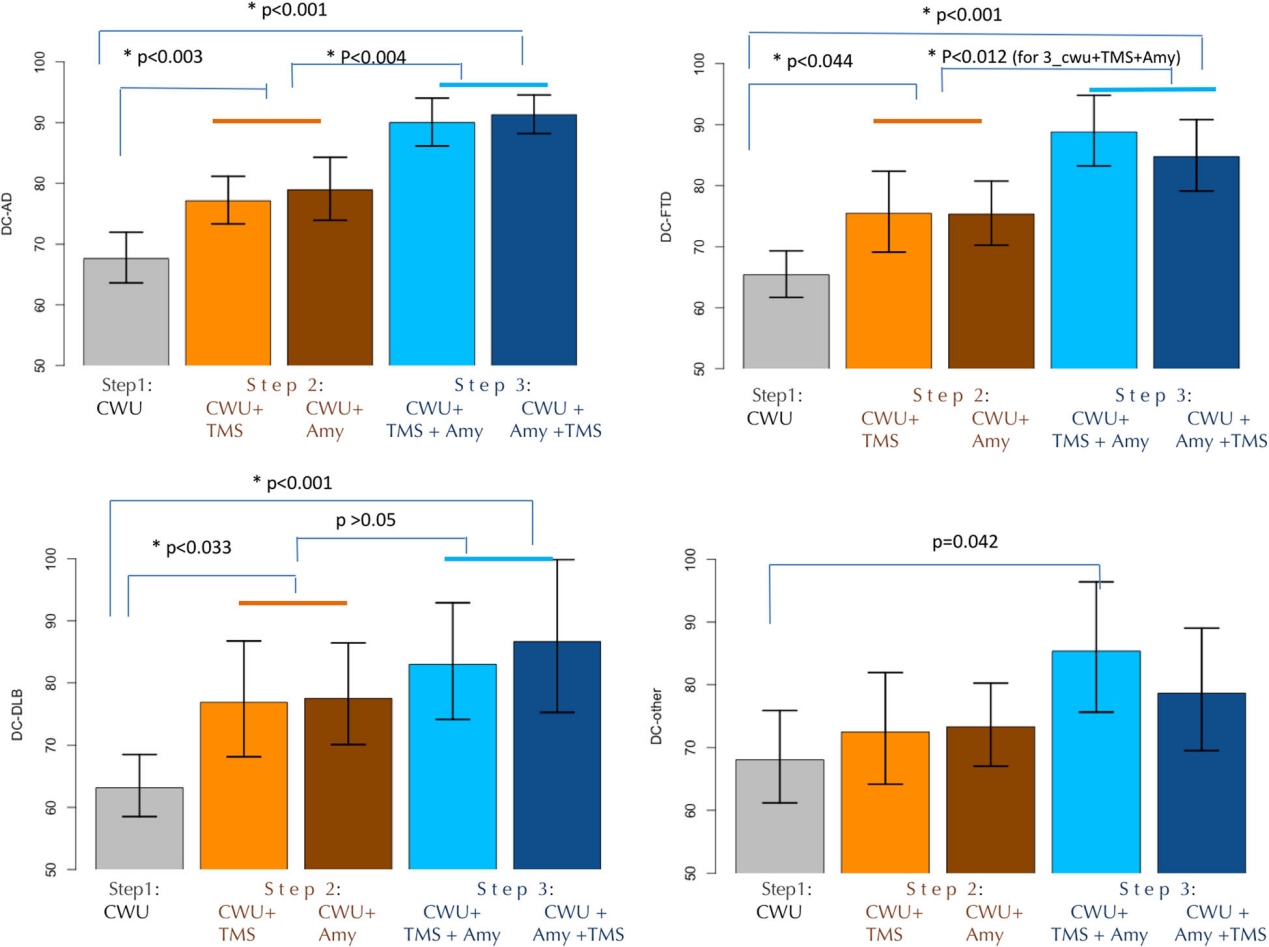
Estimates of diagnostic confidences (DCs) of different MCI subtypes at the different steps. DC-AD, diagnostic confidence of mild cognitive impairment due to Alzheimer disease; DC-FTD, diagnostic confidence of mild cognitive impairment due to frontotemporal dementia; DC-DLB, diagnostic confidence of mild cognitive impairment due to dementia with Lewy bodies; DC-other, diagnostic confidence of mild cognitive impairment due to other conditions; Cwu, clinical work-up; Cwu + TMS, clinical work-up PLUS TMS; Cwu + Amy, clinical work-up PLUS amyloid markers; Cwu + TMS + Amy, clinical work-up PLUS TMS PLUS amyloid markers; Cwu + Amy + TMS, clinical work-up PLUS amyloid markers PLUS TMS

When raters’ diagnosis was MCI-AD, the DC-AD significantly increased adding TMS (77.1, 95% CI [73.3–81.2], *p* = 0.003) or amyloid markers (78.9, 95% CI [73.9–84.3], *p* = 0.002) to clinical work-up (67.6, 95% CI [63.6–71.9]). The DC-AD further increased by considering both diagnostic markers (clinical work-up PLUS TMS PLUS amyloid markers, 90.0, 95% CI [86.2–94.1], or clinical work-up PLUS amyloid markers PLUS TMS, 91.3, 95% CI [88.2–94.6]), as compared to clinical work-up (*p* < 0.001 for both) or as compared to clinical work-up PLUS single markers (*p* = 0.004 for both TMS and amyloid markers) (see Fig. 2).

When raters’ diagnosis was MCI-FTD, the DC-FTD significantly increased adding TMS (75.5, 95% CI [69.1–82.4], *p* = 0.044) or amyloid markers (75.3, 95% CI [70.3–80.7], *p* = 0.028) to clinical work-up (65.4, 95% CI [61.7–69.3]). The DC-FTD further increased by considering both diagnostic markers (clinical work-up PLUS TMS PLUS amyloid markers, 88.8, 95% CI [83.2–94.8], or clinical work-up PLUS amyloid markers PLUS TMS, 84.8, 95% CI [79.1–90.9]) as compared to clinical work-up (*p* < 0.001 for both) or as compared to clinical work-up PLUS single markers (*p* < 0.012 only clinical work-up PLUS amyloid markers PLUS TMS vs. single markers, *p* < 0.012) (see Fig. 2).

When raters’ diagnosis was MCI-DLB, the DC-DLB significantly increased adding TMS (76.9, 95% CI [68.1–86.8], *p* = 0.033) or amyloid markers (77.5, 95% CI [70.1–86.5] *p* = 0.014) to clinical work-up alone (63.2, 95% CI [58.5–68.5]). The DC-DLB further increased by considering both diagnostic markers (clinical work-up PLUS TMS PLUS amyloid markers, 83.0, 95% CI [74.2–92.9], or clinical work-up PLUS amyloid markers PLUS TMS, 86.7, 95% CI [75.3–99.9]), as compared to clinical work-up alone (*p* < 0.001 for both), while it did not show a statistically significant difference as compared to clinical work-up PLUS single markers (*p* > 0.05 for all 4 comparisons) (see Fig. 2).

Finally, when raters’ diagnosis was MCI-other, although the DC-other increased adding TMS (72.5, 95% CI [64.2–81.9]) or amyloid markers (73.3, 95% CI [67.0–80.2]) to clinical work-up alone (68.0, 95% CI [61.2–75.9]), the increase was not statistically significant. Similarly, the DC-other further increased by considering both diagnostic markers (clinical work-up PLUS TMS PLUS amyloid markers, 85.4, 95% CI [75.6–96.4], or clinical work-up PLUS amyloid markers PLUS TMS, 78.6, 95% CI [69.5–89.0]), as compared to clinical work-up or as compared to clinical work-up PLUS single markers, but none of these was statistically significant (see Fig. 2).

For all MCI subgroups, no significant differences between the add-on effect of TMS vs. the add-on effect of amyloid markers to clinical work-up were observed (*p* > 0.732 for all four MCI diagnoses).

### Performance of each assessment section in predicting the “gold-standard” diagnosis

According to the “gold-standard” diagnosis, 48 MCI-AD (mean age ± SD 69.9 ± 7.0; female 50.0%; MMSE 26.3 ± 2.0), 31 MCI-FTD (mean age ± SD 66.8 ± 7.5; female 45.2%; MMSE 26.8 ± 2.3), 9 MCI-DLB (mean age ± SD 72.4 ± 4.2; female 66.7%; MMSE 24.7 ± 2.0), and 16 MCI-other (mean age ± SD 67.6 ± 7.9; female 50%; MMSE 27.1 ± 1.6) were included.

In Additional file 3: Table S1, cerebrospinal fluid analyses, amyloid imaging, and TMS parameter findings in MCI subtypes according to “gold standard” diagnosis were reported.

Logistic regression models revealed a high statistically significant association between “gold standard” diagnosis and all the three assessments (*p* < 0.001 for all).

**Fig. 3:**
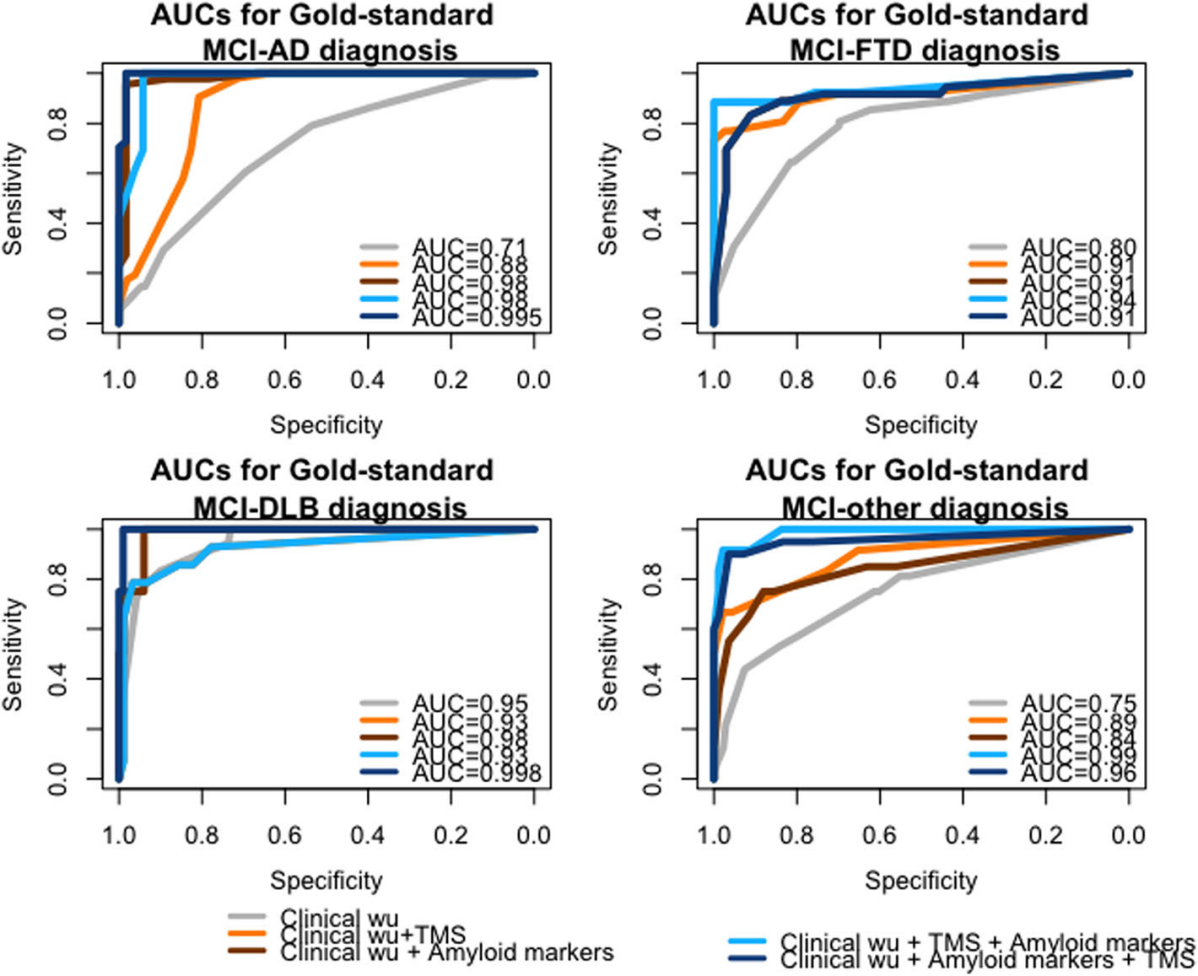
Receiver operating characteristic curve for DC-AD, DC-FTD, DC-DLB, and DC-other of each assessment section in predicting the “gold-standard” diagnosis. DC, diagnostic confidence; MCI-AD, mild cognitive impairment due to Alzheimer disease; MCI-FTD, mild cognitive impairment due to frontotemporal dementia; MCI-DLB, mild cognitive impairment due to dementia with Lewy bodies; MCI-other, mild cognitive impairment due to other conditions; Clinical wu, clinical work-up; TMS, transcranial magnetic stimulation parameters; ROC, receiver operating characteristic; AUC, area under the curve; TMS, transcranial magnetic stimulation

Considering the performance in predicting diagnosis, although all three assessments reached high values of specificity and sensitivity in classifying MCI-subtypes correctly (AUC greater than 0.7 for all), the best performances were obtained when both markers were disclosed for all MCI diagnoses (see Fig. 3). However, as reported in Table 1, amyloid markers performed better as compared to TMS in predicting MCI-AD diagnosis. Prediction of MCI-FTD diagnosis was significantly improved by the use of one single marker (either TMS or amyloid marker) as compared to clinical work-up alone, while it did not significantly improve with the add-on of a second marker. Prediction of MCI-DLB obtained good performances with clinical work-up, while predicting MCI-other diagnosis required the disclosure of both markers to achieve high accuracy.

**Table 1:** Association and performance evaluation of DC-AD, DC-FTD, DC-DLB, and DC-other of each assessment section in predicting the corresponding “gold-standard” diagnosis

Assessment steps	AUC (95%CI)	OR (95%CI)	*p* values*
Diagnosis of MCI-AD			
1. Clinical work-up	0.706 (0.637–0.775)	1.04 (1.02–1.05)	–
2. Clinical work-up PLUS TMS	0.879 (0.809–0.950)	1.07 (1.05–1.10)	1 vs. 2, *p* < 0.001
3. Clinical work-up PLUS amyloid markers	0.981 (0.953–1)	1.14 (1.09–1.22)	1 vs. 3, *p* < 0.001; 2 vs. 3, *p* < 0.010
4. Clinical work-up PLUS TMS PLUS amyloid markers	0.975 (0.946–1)	1.10 (1.07–1.16)	4 vs. 1, *p* < 0.001; 4 vs. 2, *p* < 0.001
5. Clinical work-up PLUS amyloid markers PLUS TMS	0.995 (0.986–1)	1.15 (1.09–1.38)	5 vs. 1, *p* < 0.001; 5 vs. 2, *p* < 0.001
Diagnosis of MCI-FTD			
1. Clinical work-up	0.796 (0.728–0.864)	1.05 (1.03–1.06)	–
2. Clinical work-up PLUS TMS	0.913 (0.831–0.995)	1.08 (1.06–1.12)	1 vs. 2, *p* = 0.032
3. Clinical work-up PLUS amyloid markers	0.910 (0.840–0.979)	1.07 (1.05–1.10)	3 vs. 1, *p* = 0.022
4. Clinical work-up PLUS TMS PLUS amyloid markers	0.940 (0.868–1)	1.10 (1.06–1.16)	4 vs. 1, *p* = 0.005
5. Clinical work-up PLUS amyloid markers PLUS TMS	0.910 (0.840–0.979)	1.10 (1.07–1.14)	5 vs. 1, *p* = 0.022
Diagnosis of MCI-DLB			
1. Clinical work-up	0.946 (0.907–0.946)	1.08 (1.06–1.11)	–
2. Clinical work-up PLUS TMS	0.925 (0.836–1)	1.07 (1.04–1.10)	–
3. Clinical work-up PLUS amyloid markers	0.983 (0.951–1)	1.08 (1.04–1.16)	–
4. Clinical work-up PLUS TMS PLUS amyloid markers	0.925 (0.836–1)	1.07 (1.05–1.11)	–
5. Clinical work-up PLUS amyloid markers PLUS TMS	0.998 (0.991–1)	1.33 (1.06–1.50)	–
Diagnosis of MCI-other			
1. Clinical work-up	0.752 (0.652–0.848)	1.04 (1.02–1.06)	–
2. Clinical work-up PLUS TMS	0.889 (0.772–1)	1.08 (1.05–1.12)	–
3. Clinical work-up PLUS amyloid markers	0.844 (0.729–0.959)	1.06 (1.04–1.08)	–
4. Clinical work-up PLUS TMS PLUS amyloid markers	0.986 (0.965–1)	1.09 (1.06–1.15)	4 vs. 1, *p* < 0.001
5. Clinical work-up PLUS amyloid markers PLUS TMS	0.957 (0.895–1)	1.09 (1.06–1.13)	5 vs. 1, *p* < 0.001

Association was evaluated by odds ratio (OR) of logistic regression models; the performance was evaluated through AUC values of the receiver operating characteristic (ROC) curves

*DC* diagnostic confidence, *MCI-AD* mild cognitive impairment due to Alzheimer disease, *MCI-FTD* mild cognitive impairment due to frontotemporal dementia, *MCI-DLB* mild cognitive impairment due to dementia with Lewy bodies, *MCI-other* mild cognitive impairment due to other conditions, *AUC* area under the curve, *CI* confidence interval, *TMS* transcranial magnetic stimulation

*AUC comparisons: *p* value of significantly different AUC

## Discussion

This study has shown that the addition of such TMS markers to clinical work-up, such as SAI and SICI-ICF measures, significantly increased the diagnostic confidence of MCI patients compared to clinical work-up alone. Moreover, there was no statistical evidence of difference between the add-on effect of TMS markers and amyloid-related markers, and between the sequences of presentation of TMS/amyloid findings, while the diagnostic confidence further increased when both markers were available. An unexpected effect was observed when the clinical expertise was taken into account, as the greater the clinician’s experience, the greater the flexibility in considering alternative diagnoses and in modifying diagnostic confidence by using available markers.

The clinical diagnosis of AD is still generally based on an extensive evaluation of cognitive and behavioral performance, along with functional status, which provides a variable grade of accuracy, with a definite diagnosis reached only at autopsy [31]. Because it is generally considered that disease-modifying treatments are likely to be most effective at the earliest stages of AD, there is great effort to develop sensitive markers that facilitate detection and monitoring of early brain changes in at-risk individuals. Many technological advancements have been implemented to serve as surrogates for specific neuropathological hallmarks and to improve the diagnostic work-up of cognitive decline [32].

Indeed, it has been clearly demonstrated that the development of AD pathology, as measured by PET amyloid or CSF amyloid marker positivity, can start 20 to 30 years before dementia onset, implying that there is a wide window of opportunities to start a preventive treatment [33]. Thus, growing evidence has emerged arguing for staging AD along a continuum by means of surrogate markers of amyloid burden, which have been used to improve the diagnostic confidence of AD [31]. Accordingly, in a recent naturalistic study, positron emission tomography amyloid data led to improved confidence in 81.5% of patients with complex dementia presentation and altered management in 80.0% of cases [34].

If amyloid markers held the premises to identify or to exclude preclinical AD [35], a number of issues need to be further elucidated, especially in MCI stages. Cerebrospinal fluid analysis is invasive and needs hospitalization, and although good sensitivity in diagnosing preclinical AD [36], it is not helpful in the differential diagnosis among MCI non-AD subtypes [37]. Moreover, there is still variability in cerebrospinal fluid measurements between clinical laboratories and between batches of reagents, which are more pronounced for Aβ42 dosages [38]. Positron emission tomography amyloid has been shown to have high sensitivity and specificity for brain amyloidosis, and not necessarily for AD, particularly in the elderly population, thus resulting more useful as an exclusion criteria for AD [39], and besides being expensive, it is still not available in most dementia centers and not reimbursed in most western countries.

In this context, we have recently proposed TMS intracortical connectivity markers, which assess neurotransmitter deficits [15, 40], instead of targeting surrogate neuropathological hallmarks, and we obtained comparable findings in defining diagnostic accuracy and diagnostic confidence in MCI subjects.

TMS has a number of advantages as compared to amyloid markers, even though its use is still limited in selected centers. TMS is time-saving, non-invasive, and inexpensive, and it can ideally be performed during the patient’s first access to the clinic, allowing the clinician to identify subjects deserving further in-depth examinations. Interestingly, the most experienced clinician used the markers more profitably, with a greater ability in interpreting diagnostic markers results. Furthermore, the highest diagnostic confidence (Fig. 2) and the highest diagnostic accuracy (Table 1) were reached when both markers were disclosed, confirming that markers used in combination may best identify prodromal AD, prodromal FTD, or prodromal DLB [32]. In this view, TMS parameters may be acknowledged among the wide variety of available markers for AD and for other dementias, such as functional and structural neuroimaging methods, molecular techniques based on CSF, and blood analyses, and may be considered as an add-on marker to be used in combination to increase diagnostic confidence. The role of additional biomarkers, such as TMS, could become useful particularly in cases with contrasting biomarkers of neurodegeneration or amyloidosis obtained from different techniques, and in cases where these biomarkers are unavailable, or contraindicated in the single patient. Furthermore, considering the high sensitivity of the technique (90–95%), the test could be particularly suitable to be used as a screening tool in the initial diagnostic assessment, both for confirming the presence of a dementing illness and for differentiating distinct neurodegenerative disorders, thus helping in the decision for the most appropriate clinical work-up.

Some limitations of the present study need to be acknowledged. First, longitudinal follow-up of included subjects is needed to clearly prove the usefulness of both amyloid markers and TMS measures. Second, we conducted a retrospective study using medical records; thus, the evaluation of the add-on value of TMS parameters should be further addressed in real-world situations. Third, in this preliminary work, we considered only two raters, and it might be worth having more raters to further address diagnostic accuracy.

## Conclusions

In MCI subjects, TMS parameters are useful as an add-on marker in addition to routine clinical assessment and may be considered in combination with amyloid markers to reach the highest diagnostic accuracy and confidence on clinical grounds.

Longitudinal follow-up studies on larger samples of subjects referred for cognitive impairment aimed to compare the predictive values of TMS parameters as well as of other well-known amyloid and neurodegenerative biomarkers might be helpful to warrant the inclusion of TMS in the diagnostic algorithm of neurodegenerative dementias.

## Supplementary information

**Additional file 1: Figure S1.** Diagnostic Confidence (DC) descriptive statistics and GEE results on overall diagnoses. Blue bullet: GEE estimated mean values; blue vertical line: GEE estimated 95% Wald’s Confidence Interval for the mean; black triangle: median values. DC-AD: Diagnostic Confidence of Mild Cognitive Impairment due to Alzheimer Disease; DC-FTD: Diagnostic Confidence of Mild Cognitive Impairment due to Frontotemporal dementia; DC-DLB: Diagnostic Confidence of Mild Cognitive Impairment due to Dementia with Lewy Bodies; DC-other: Diagnostic Confidence of Mild Cognitive Impairment due to other conditions.

**Additional file 2: Figure S2.** Estimated mean (points) and corresponding 95% CI (vertical bars) of the DCs in the three assessment steps. Step 1: Clinical work-up (Cwu); Step 2: Cwu + TMS (arm1) or Cwu + Amyloid markers (arm2); Step3: Cwu + TMS + Amyloid markers (arm1) or Cwu + Amyloid markers+TMS (arm2).

**Additional file 3: Table S1.** Cerebrospinal fluid markers, amyloid PET imaging and TMS parameters in MCI subtypes according to “gold standard” diagnosis.

## Data Availability

The datasets used and/or analyzed during the current study are available from the corresponding author on reasonable request.

## Abbreviations

AD: Alzheimer disease
DC: Diagnostic confidence
DLB: Dementia with Lewy bodies
FTD: Frontotemporal dementia
ICF: Intracortical facilitation
MCI: Mild cognitive impairment
SAI: Short-latency afferent inhibition
SICI: Short-interval intracortical inhibition
TMS: Transcranial magnetic stimulation

## References

[1] Petersen RC, Lopez O, Armstrong MJ (2018). Practice guideline update summary: mild cognitive impairment. Neurology.

[2] Price JL, McKeel DW, Buckles VD (2009). Neuropathology of nondemented aging: presumptive evidence for preclinical Alzheimer disease. Neurobiol Aging.

[3] Bennett DA, Schneider JA, Arvanitakis Z (2006). Neuropathology of older persons without cognitive impairment from two community-based studies. Neurology.

[4] Albert MS, Dekosky ST, Dickson D (2011). The diagnosis of mild cognitive impairment due to Alzheimer’s disease: recommendations from the National Institute on Aging-Alzheimer’s Association workgroups on diagnostic guidelines for Alzheimer’s disease. JALZ.

[5] Shaw LM, Vanderstichele H, Knapik-Czajka M (2009). Cerebrospinal fluid biomarker signature in Alzheimer’s disease neuroimaging initiative subjects. Ann Neurol.

[6] Clark CM, Pontecorvo MJ, Beach TG (2012). Cerebral PET with florbetapir compared with neuropathology at autopsy for detection of neuritic amyloid-β plaques: a prospective cohort study. Lancet Neurol.

[7] de Souza LC, Lamari F, Belliard S (2011). Cerebrospinal fluid biomarkers in the differential diagnosis of Alzheimer’s disease from other cortical dementias. J Neurol Neurosurg Psychiatry.

[8] Ikonomovic MD, Klunk WE, Abrahamson EE (2008). Post-mortem correlates of in vivo PiB-PET amyloid imaging in a typical case of Alzheimer’s disease. Brain.

[9] Boccardi M, Altomare D, Ferrari C (2016). Assessment of the incremental diagnostic value of florbetapir F 18 imaging in patients with cognitive impairment. JAMA Neurol.

[10] Visser PJ, Verhey F, Knol DL (2009). Prevalence and prognostic value of CSF markers of Alzheimer’s disease pathology in patients with subjective cognitive impairment or mild cognitive impairment in the DESCRIPA study: a prospective cohort study. Lancet Neurol.

[11] Benussi A, Di Lorenzo F, Dell’Era V (2017). Transcranial magnetic stimulation distinguishes Alzheimer disease from frontotemporal dementia. Neurology.

[12] Murley AG, Rowe JB (2018). Neurotransmitter deficits from fronto temporal lobar degeneration. Brain.

[13] Tarawneh R, Galvin JE (2007). Distinguishing Lewy body dementias from Alzheimer’s disease. Expert Rev Neurother.

[14] Khundakar AA, Hanson PS, Erskine D (2016). Analysis of primary visual cortex in dementia with Lewy bodies indicates GABAergic involvement associated with recurrent complex visual hallucinations. Acta Neuropathol Commun.

[15] Rossini PM, Burke D, Chen R (2015). Non-invasive electrical and magnetic stimulation of the brain, spinal cord, roots and peripheral nerves: basic principles and procedures for routine clinical and research application. An updated report from an I.F.C.N. Committee. Clin. Neurophysiol..

[16] Padovani A, Benussi A, Cantoni V (2018). Diagnosis of mild cognitive impairment due to Alzheimer’s disease with transcranial magnetic stimulation. J Alzheimers Dis.

[17] Benussi A, Cosseddu M, Filareto I (2016). Impaired long-term potentiation-like cortical plasticity in presymptomatic genetic frontotemporal dementia. Ann Neurol.

[18] Benussi A, Gazzina S, Premi E (2019). Clinical and biomarker changes in presymptomatic genetic frontotemporal dementia. Neurobiol Aging.

[19] Benussi A, Dell’Era V, Cantoni V (2018). Discrimination of atypical parkinsonisms with transcranial magnetic stimulation. Brain Stimul.

[20] Benussi A, Alberici A, Ferrari C (2018). The impact of transcranial magnetic stimulation on diagnostic confidence in patients with Alzheimer disease. Alzheimers Res Ther.

[21] Magni E, Binetti G, Bianchetti A (1996). Mini-mental state examination: a normative study in Italian elderly population. Eur J Neurol.

[22] Morris JC (1993). The clinical dementia rating (CDR): current version and scoring rules. Neurology.

[23] Katz S, Ford AB, Moskowitz RW (1963). Studies of illness in the aged the index of ADL: a standardized measure of biological and psychosocial function. JAMA.

[24] Lawton MP, Brody EM (1969). Assessment of older people: self-maintaining and instrumental activities of daily living. Gerontologist.

[25] Cummings JL, Mega M, Gray K (1994). The neuropsychiatric inventory: comprehensive assessment of psychopathology in dementia. Neurology.

[26] Galeoto G, Sansoni J, Scuccimarri M (2018). A psychometric properties evaluation of the Italian version of the geriatric depression scale. Depress Res Treat.

[27] Borroni B, Benussi A, Archetti S (2015). Csf p-tau181/tau ratio as biomarker for TDP pathology in frontotemporal dementia. Amyotroph Lateral Scler Front Degener.

[28] Kujirai T, Caramia MD, Rothwell JC (1993). Corticocortical inhibition in human motor cortex. J Physiol.

[29] Ziemann U, Rothwell JC, Ridding MC (1996). Interaction between intracortical inhibition and facilitation in human motor cortex. J Physiol.

[30] Tokimura H, Di Lazzaro V, Tokimura Y (2000). Short latency inhibition of human hand motor cortex by somatosensory input from the hand. J. Physiol.

[31] Jack CR, Bennett DA, Blennow K (2018). NIA-AA research framework: toward a biological definition of Alzheimer’s disease. Alzheimers Dement.

[32] Frisoni GB, Boccardi M, Barkhof F (2017). Strategic roadmap for an early diagnosis of Alzheimer’s disease based on biomarkers. Lancet Neurol.

[33] Jansen WJ, Ossenkoppele R, Knol DL (2015). Prevalence of cerebral amyloid pathology in persons without dementia: a meta-analysis. JAMA.

[34] Ceccaldi M, Jonveaux T, Verger A (2018). Added value of 18F-florbetaben amyloid PET in the diagnostic workup of most complex patients with dementia in France: a naturalistic study. Alzheimers Dement.

[35] Sperling RA, Aisen PS, Beckett LA (2011). Toward defining the preclinical stages of Alzheimer’s disease: recommendations from the National Institute on Aging-Alzheimer’s Association workgroups on diagnostic guidelines for Alzheimer’s disease. JALZ.

[36] Bos I, Vos S, Verhey F (2019). Cerebrospinal fluid biomarkers of neurodegeneration, synaptic integrity, and astroglial activation across the clinical Alzheimer’s disease spectrum. Alzheimers Dement.

[37] Paterson RW, Slattery CF, Poole T (2018). Cerebrospinal fluid in the differential diagnosis of Alzheimer’s disease: clinical utility of an extended panel of biomarkers in a specialist cognitive clinic. Alzheimers Res Ther.

[38] Mattsson N, Andreasson U, Persson S (2011). The Alzheimer’s Association external quality control program for cerebrospinal fluid biomarkers. Alzheimer’s Dement.

[39] Ossenkoppele R, Jansen WJ, Rabinovici GD (2015). Prevalence of amyloid PET positivity in dementia syndromes: a meta-analysis. JAMA.

[40] Ziemann U, Reis J, Schwenkreis P (2015). TMS and drugs revisited 2014. Clin Neurophysiol.

