# Supplementary figures and images for: Transcranial magnetic stimulation and amyloid markers in mild cognitive impairment: impact on diagnostic confidence and diagnostic accuracy
Source: Alzheimers Res Ther. 2019 Dec 1;11:95. doi: 10.1186/s13195-019-0555-3 (PMC6886207; doi:10.1186/s13195-019-0555-3)

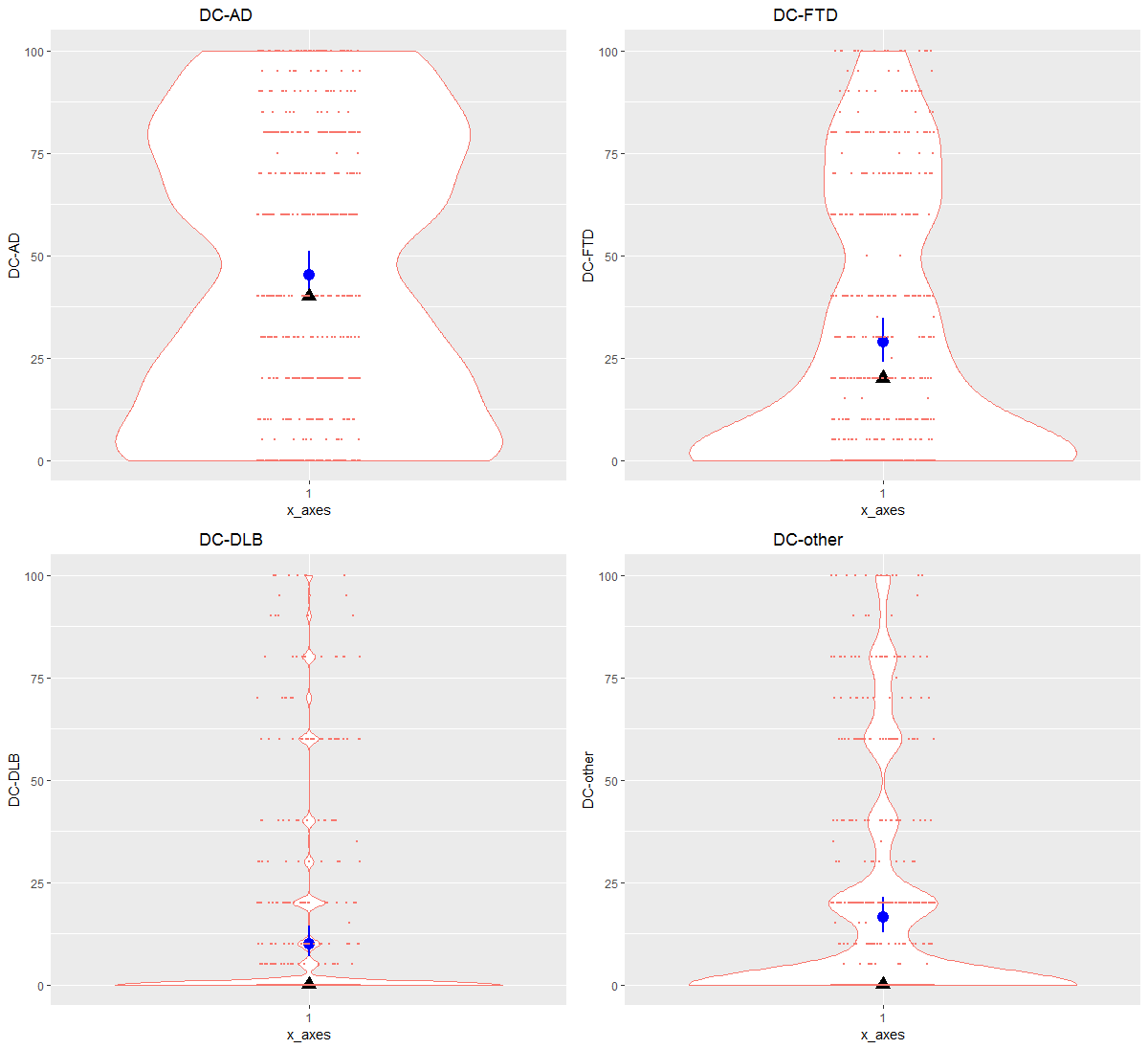

Supplement: Supplementary file 1 — Additional file 1: Figure S1. Diagnostic Confidence (DC) descriptive statistics and GEE results on overall diagnoses. Blue bullet: GEE estimated mean values; blue vertical line: GEE estimated 95% Wald’s Confidence Interval for the mean; black triangle: median values. DC-AD: Diagnostic Confidence of Mild Cognitive Impairment due to Alzheimer Disease; DC-FTD: Diagnostic Confidence of Mild Cognitive Impairment due to Frontotemporal dementia; DC-DLB: Diagnostic Confidence of Mild Cognitive Impairment due to Dementia with Lewy Bodies; DC-other: Diagnostic Confidence of Mild Cognitive Impairment due to other conditions. [file 13195_2019_555_MOESM1_ESM.tiff]

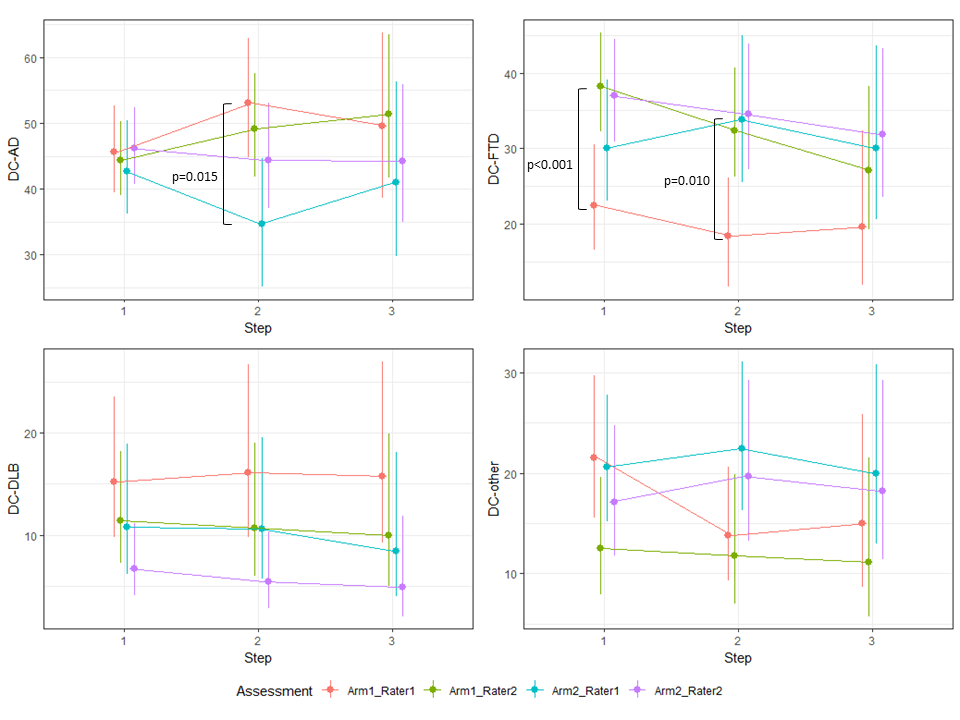

Supplement: Supplementary file 2 — Additional file 2: Figure S2. Estimated mean (points) and corresponding 95% CI (vertical bars) of the DCs in the three assessment steps. Step 1: Clinical work-up (Cwu); Step 2: Cwu + TMS (arm1) or Cwu + Amyloid markers (arm2); Step3: Cwu + TMS + Amyloid markers (arm1) or Cwu + Amyloid markers+TMS (arm2). [file 13195_2019_555_MOESM2_ESM.tif]
