# Supplementary material for: Transcranial magnetic stimulation and amyloid markers in mild cognitive impairment: impact on diagnostic confidence and diagnostic accuracy
Source: Alzheimers Res Ther. 2019 Dec 1;11:95. doi: 10.1186/s13195-019-0555-3 (PMC6886207; doi:10.1186/s13195-019-0555-3)
Supplement: Supplementary file 3 — Additional file 3: Table S1. Cerebrospinal fluid markers, amyloid PET imaging and TMS parameters in MCI subtypes according to “gold standard” diagnosis. [file 13195_2019_555_MOESM3_ESM.docx]

**Supplementary materials**

**Supplementary Table S1. Cerebrospinal fluid markers, amyloid PET imaging and TMS parameters in MCI subtypes according to “gold standard” diagnosis.**

| Variable | **MCI-AD** | **MCI-FTD** | **MCI-DLB** | **MCI-other** | p* |
| --- | --- | --- | --- | --- | --- |
| **CSF markers** |  |  |  |  |  |
| Aβ_1-42_, pg/mL | 546.6±175.7 | 969.0±251.1 | 934.0±258.5 | 1162.3±398.4 | 0.001 |
| Tau, pg/mL | 696.7±449.9 | 310.0±149.0 | 179.6±42.3 | 199.0±64.5 | 0.001 |
| Phospho-Tau, pg/mL | 86.0±35.6 | 50.1±34.0 | 35.8±5.8 | 42.7±13.1 | 0.001 |
| **Amyloid PET positive, n** | 34/35 | 0/10 | 1/4 | 0/10 | 0.001^ |
| **TMS parameters** |  |  |  |  |  |
| SAI | 0.83±0.12 | 0.58±0.11 | 0.98±0.10 | 0.55±0.09 | 0.001 |
| SICI-ICF | 0.29±0.19 | 0.81±0.28 | 0.70±0.23 | 0.22±0.12 | 0.001 |
| SICI-ICF/SAI ratio | 0.36±0.31 | 1.54±0.62 | 0.72±0.24 | 0.40±0.25 | 0.001 |

*p-values of One-way ANOVA among groups unless otherwise specified; ^Chi-Square test.

MCI-AD: Mild Cognitive Impairment due to Alzheimer Disease; MCI-FTD: Mild Cognitive Impairment due to Frontotemporal dementia; MCI-DLB: Mild Cognitive Impairment due to Dementia with Lewy Bodies; MCI-other: Mild Cognitive Impairment due to other conditions.

CSF: cerebrospinal fluid; SAI: mean short-latency afferent inhibition (0, ^+^4 ms); SICI-ICF: mean short-interval intracortical inhibition (1, 2, 3 ms) / intracortical facilitation (7, 10, 15 ms); SICI-ICF/SAI = ratio between SICI-ICF and SAI parameters.

**Figure S1. Diagnostic Confidence (DC) descriptive statistics and GEE results on overall diagnoses.**

Blue bullet: GEE estimated mean values; blue vertical line: GEE estimated 95% Wald’s Confidence Interval for the mean; black triangle: median values.

DC-AD: Diagnostic Confidence of Mild Cognitive Impairment due to Alzheimer Disease; DC-FTD: Diagnostic Confidence of Mild Cognitive Impairment due to Frontotemporal dementia; DC-DLB: Diagnostic Confidence of Mild Cognitive Impairment due to Dementia with Lewy Bodies; DC-other: Diagnostic Confidence of Mild Cognitive Impairment due to other conditions.

**Figure S2. Estimated mean (points) and corresponding 95% CI (vertical bars) of the DCs in the three assessment steps.**

Step 1: Clinical work-up (Cwu); Step 2: Cwu+TMS (arm1) or Cwu+Amyloid markers (arm2); Step3: Cwu+TMS+Amyloid markers (arm1) or Cwu+Amyloid markers+TMS (arm2).
